# The importance of SHBG and calculated free testosterone for the diagnosis of symptomatic hypogonadism in HIV-infected men: a single-centre real-life experience

**DOI:** 10.1007/s15010-020-01558-6

**Published:** 2020-12-08

**Authors:** Letizia Chiara Pezzaioli, Eugenia Quiros-Roldan, Simone Paghera, Teresa Porcelli, Filippo Maffezzoni, Andrea Delbarba, Melania Degli Antoni, Carlo Cappelli, Francesco Castelli, Alberto Ferlin

**Affiliations:** 1 Department of Clinical and Experimental Sciences, Unit of Endocrinology and Metabolism, University of Brescia, Viale Europa 11, 25123 Brescia, Italy; 2 Department of Clinical and Experimental Sciences, Unit of Infectious and Tropical Diseases, University of Brescia and ASST Spedali di Brescia, Brescia, Italy; 3 Centro Di Ricerca Emato-Oncologica AIL (CREA), Diagnostic Department, ASST Spedali Civili di Brescia, Brescia, Italy; 4 Endocrinology, Montichiari Hospital, ASST Spedali Civili Brescia, Montichiari, Italy; 5 Unit of Endocrinology and Metabolism, Department of Medicine, ASST Spedali Civili Brescia, Brescia, Italy

**Keywords:** HIV, Hypogonadism, Calculated free testosterone, SHBG, Gonadotropins

## Abstract

**Purpose:**

The prevalence of low testosterone and symptoms of hypogonadism in HIV-infected men is still debated. We aimed to estimate the prevalence and type of hypogonadism in HIV-infected males complaining about sexual symptoms, and to evaluate the role of calculated free testosterone (cFT) *vs* total testosterone (TT) for diagnosis. Furthermore, we evaluated relationship between sex hormone-binding globulin (SHBG), gonadal status and clinical and virologic parameters.

**Methods:**

We retrospectively evaluated 169 HIV-infected men with sexual symptoms, with TT available. Among them, we selected 94 patients with TT, SHBG, cFT, and luteinizing hormone (LH) available, and classified hypogonadism into overt (low TT and/or low cFT) and compensated (high LH, normal TT and cFT). Comparison was performed by non-parametric Kruskal–Wallis test and Spearman’s correlation was calculated to verify the possible associations.

**Results:**

Overt and compensated hypogonadism were found in 20.2% and 13.8% of patients, respectively. With reliance on TT alone, only 10.6% of patients would have met diagnosis. SHBG values were elevated in one third of patients, and higher in men with compensated hypogonadism. Significant positive correlation was found between SHBG and HIV infection duration, TT and LH.

**Conclusion:**

Only a complete hormonal profile can properly diagnose and classify hypogonadism in HIV-infected men complaining about sexual symptoms. TT alone reliance may lead to half of diagnoses missing, while lack of gonadotropin prevents the identification of compensated hypogonadism. This largely comes from high SHBG, which seems to play a central role in the pathogenesis of hypogonadism in this population.

## Introduction

Since the early stages of HIV epidemic, hypogonadism has been recognized as a known frequent associated condition. Hypogonadism is a clinical syndrome caused by failure of the testis to produce physiological amounts of testosterone and/or a normal number of spermatozoa, depending on alteration at different levels of the hypothalamic–pituitary–testicular axis [1]. Importantly, male hypogonadism diagnosis can be done only when consistently low morning testosterone concentrations are combined with clinical manifestations of testosterone deficiency [1–3], such as specific sexual symptoms like reduced libido, erectile dysfunction (ED), decreased spontaneous erections, and less specific signs and symptoms (loss of body/facial hair, decreased testicular volume, increased body fat/reduced muscle mass, central obesity, osteoporosis, asthenia, decreased concentration, etc.) [3]. Even if cut-off values for lower limit of testosterone levels are not uniform in the different guidelines [3, 4], generally hypogonadism is considered very likely for total testosterone (TT) levels < 2.31 ng/ml and highly unlikely for TT levels > 3.46 ng/ml. For borderline levels between 2.31 and 3.46 ng/ml and in conditions with altered sex hormone-binding globulin (SHBG) levels (e.g., ageing, hyperthyroidism, liver disease, HIV infection), calculated free testosterone (cFT) determination could be helpful to reach a diagnosis [3], although standard cutoff isn’t well defined. Moreover, determination of luteinizing hormone (LH) levels allows distinction between primary hypogonadism, with low TT levels and high gonadotropins, due to a direct testicular damage, and secondary forms, with low to normal gonadotropins, due to defects in hypothalamic–pituitary–testis axis [1]. Elevated LH with normal TT levels identifies the so-called compensated hypogonadism.

Although the association between HIV and hypogonadism is well known [5], the exact prevalence of hypogonadism is difficult to calculate, due to both the heterogeneity of the diagnostic criteria and to the overlapping of hypogonadism symptoms with the infection itself. Notwithstanding this, a commonly accepted estimate of hypogonadism prevalence before the introduction of combined antiretroviral therapy (cART) ranges from 30 to over 40% [6]. With cART introduction, a reduction in the prevalence of hypogonadism has been reported, even if it is not possible to clearly establish whether this decrease is true, because data from the pre-cART and cART period are not directly comparable, since in the pre-cART era specific cutoff values for lower limits of testosterone were not available, even in general population, and also in the cART era differences in analytical methods and threshold values of testosterone still exist [5]. Nevertheless, among the studies in agreement with guidelines regarding biochemical diagnosis of hypogonadism [7–13], a cumulative prevalence of about 20% is found.

Despite many authors have already investigated the prevalence of hypogonadism in HIV-infected men, some possible limitations must be considered. First of all, clinical symptoms of hypogonadism were rarely considered as diagnostic criteria, and most of the papers focused only on testosterone values to make diagnosis [5]. Moreover, most of the studies did not measure gonadotropins, as already pointed out by Rochira and colleagues [14], thus making difficult to understand the pathogenesis of hormonal deficit in this population. Finally, albeit even Infectious Disease guidelines [15] suggest the assessment of cFT in HIV population, due to the risk of underestimation when reliance is set on TT alone [8], many studies did not measure it, or used inaccurate assays [6] for free testosterone measurement.

Therefore, we aimed to estimate the prevalence of true hypogonadism (clinical and biochemical) in HIV-infected males complaining about sexual symptoms, using different and gradually more specific biochemical criteria, to classify hypogonadism according to gonadotropin levels and evaluate the rate of misdiagnosis resulting from the use of TT alone versus a complete hormonal profile, including cFT and LH measurement. Furthermore, we aimed to evaluate possible relationship between gonadal status and some clinical and virological parameters.

## Methods

An observational retrospective study was performed. We assessed for inclusion 189 HIV-infected males, sent for symptoms of hypogonadism from HIV-specialist to the outpatient clinic of Endocrinology over a period from 2012 to 2019.

Inclusion criteria were: age > 18 years, serologically documented HIV infection in stable condition under cART and presence of self-referred sexual symptoms (reduced libido, ED, decreased spontaneous erections), the availability of HIV infection parameters (time since HIV diagnosis, cART duration and CD4 nadir count—absolute and percentual), and blood samples for hormonal parameters carried out at central hospital laboratory. Patients treated with androgens or drugs with potential detrimental role on gonadal function were excluded, as well as those with hormonal assays not analyzed in central hospital laboratory.

**Fig. 1:**
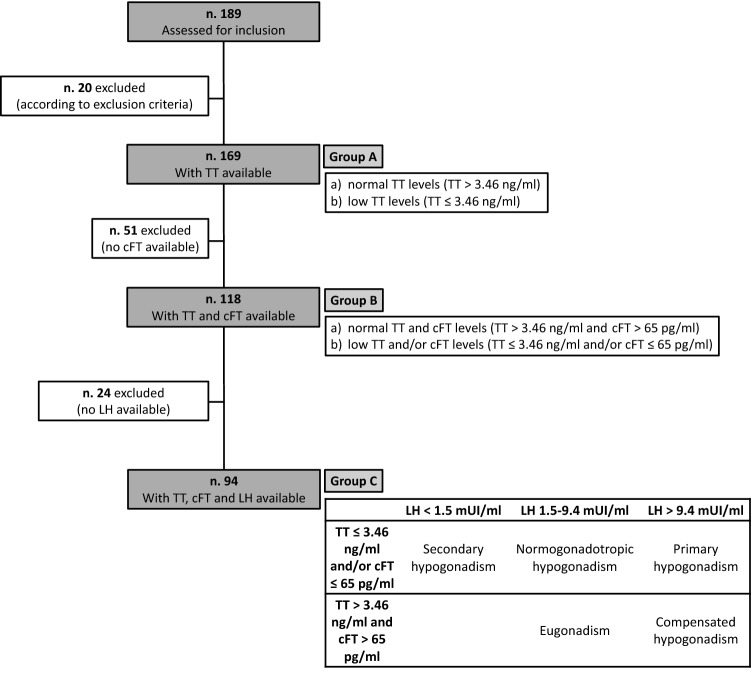
Study design. Three-step inclusion criteria

We performed three-step inclusion criteria, as shown in Fig. 1. At first, we included, following inclusion and exclusion criteria, all patients with properly measured TT available (Group A, n. 169). From this initial evaluation, we obtained a further subgroup, by considering only patients with both TT, SHBG and cFT available, to evaluate the possible contribution of the latter to the diagnosis of hypogonadism (Group B, n. 118). Finally, we obtained a further restricted subgroup, including only patients with TT, SHBG, cFT, and LH available (Group C, n. 94), to select a population with complete hormonal profiling, to better define the hypothalamic–pituitary–testicular function.

Cutoff value for lower limit of TT was set at 3.46 ng/ml; normal range for LH was established between 1.5 and 9.4 mUI/ml; cFT values were considered low if ≤ 65 pg/ml [1, 3, 16, 17], whereas SHBG was considered high when > 70 nmol/l, according to central hospital laboratory cutoffs.

Figure 1 also summarizes, for all subgroups, the classification of different forms of hypogonadism according to the aforementioned hormonal cutoffs.

All blood samples were obtained between 8.00 and 10.00 a.m., after a 12-h overnight fast, and were analyzed at central hospital laboratory (ASST Spedali Civili Brescia). TT and LH were determined using CMIA, SHBG using CLIA (chemiluminescence immunoassay). cFT was calculated with Vermeulen equation (http://www.issam.ch/freetesto.htm), that combine TT, SHBG and albumin. CD4 count was assessed by flow cytometry. Interassay coefficient of variance for TT, LH and SHBG was < 5%.

Ethical approval for this study was obtained from Local Ethical Committee (Comitato Etico di Brescia, NP 3898) and informed consent was obtained from all participants.

### Statistical analysis

GraphPad Prism version 5.1 (GraphPad Software, San Diego, CA) was used for statistical analysis. Since the variables were not normally distributed (D’Agostino and Pearson omnibus normality test was used), comparison among medians of the quantitative variables were performed by non-parametric Kruskal–Wallis *H* test, followed by post-hoc Dunn’s test when a significant difference was found. The Spearman’s correlation was calculated to verify the possible association with TT and cFT of the variables used. *P* values ≤ 0.05 were considered significant.

## Results

An overview of the clinical information of the 169 patients, grouped according to TT levels (Group A, 169 patients), TT, SHBG and cFT (Group B, 118 patients), and complete hormonal profile, including TT, SHBG, cFT and LH (Group C, 94 patients) is summarized in Table 1. All patients were in stable clinical conditions under cART, with adequate CD4 + count and viral suppression at time of investigation.

**Table 1:** Clinical characteristics and prevalence of hypogonadism in a cohort of HIV-infected outpatients with hypogonadal symptoms, according to three-step diagnostic criteria

	Group A (n. 169)	Group B (n. 118)	Group C (n. 94)
Age (yr)	53.0 (48.0–58.0)	53.0 (49.0–59.0)	53.0 (49.0–58.0)
HIV infection duration (yr)	14.0 (8.0–24.0)	14.0 (8.0–23.0)	12.0 (7.8–22.3)
cART duration (yr)	11.0 (6.0–18.0)	11.0 (6.0–18.0)	10.0 (5.0–18.0)
BMI (kg/m^2^)	25.1 (1.6–27.6)	25.0 (22.3–27.6)	25.1 (22.7–27.5)
CD4 + nadir (cell/mm^3^)	155.0 (55.3–73.8)	166.5 (63.0–277.3)	132.0 (51.5–278.0)
CD4 + nadir (%)	14.6 (7.8–22.1)	14.7 (8.8–23.0)	14.6 (8.4–22.3)
Total Testosterone (ng/ml)	6.7 (5.2–8.7)	6.8 (5.2–9.2)	6.7 (4.7–8.8)
SHBG (nmol/l)	/	64.0 (44.3–90.0)	63.0 (41.2–87.8)
calculated free Testosterone (pg/ml)	/	94.5 (72.1–116.8)	93.6 (70.2–118.5)
LH (mUI/ml)	/	/	5.6 (3.3–8.4)
Gonadal status			
n. eugonadism (%)	155 (91.7)	96 (81.4)	75 (79.8)
n. hypogonadism (%)	14 (8.3)	22 (18.6)	19 (20.2)
Primary (%)	/	/	5 (5.3)
Secondary (%)	/	/	2 (2.1)
Normogonadotropic (%)	/	/	12 (12.8)
Compensated (%)	/	/	13 (13.8)

Continuous variables are shown as median (interquartile range). The prevalence of hypogonadism, defined according to diagnostic criteria reported in Fig. 1, is presented as number (%)

When considering Group A, TT levels below 3.46 ng/ml were found in 14/169 (8.3%) patients. When also SHBG and cFT were assessed (Group B, 118 patients), overt hypogonadism was found in 22/118 (18.6%) patients. In detail, of these 22 patients, only 10 would have met diagnosis based on TT values. Therefore, the addition of cFT in Group B allowed the identification of 12 further patients, who otherwise would have been considered eugonadal, with a neat increase in diagnosis rate of 2.2 times.

We then focused our study only on patients with complete hormonal profile available, including TT, SHBG, cFT and LH (Group C, 94 patients). We found overt hypogonadism in 19/94 (20.2%) patients. The assessment of LH values allowed us to classify hypogonadism for these patients, and we found that 5/19 (26.3%) showed primary hypogonadism and 14/19 (73.7%) had secondary and/or normogonadotropic hypogonadism. Moreover, 13/94 (13.8%) patients, despite having TT and cFT within normal range, showed clearly increased LH values, fitting the definition of compensated hypogonadism, and bringing the total number of patients suffering from any gonadal axis alteration to 32/94 (34.0%).

If we had used TT alone as diagnostic criterium in these patients, as we did in Group A, only 10 out of 94 (10.6%) would have met hypogonadism diagnosis, a prevalence close to what we actually found in Group A. The addition of cFT has, therefore, allowed us to diagnose overt hypogonadism in 19 patients instead of 10, with an increase of 1.9 times; otherwise, these 9 patients with low cFT but normal TT would have been considered eugonadal or with compensated form, if considering TT alone or TT and LH.

Subsequently, a comparison among groups according to gonadal status was performed for Group C (94 patients), as shown in Table 2. We initially considered all five different categories, keeping them separated, and we found statistically significant differences for testosterone and gonadotropin values, as expected, and for HIV infection duration (data not shown). Since some subclasses of hypogonadism had a small sample size, we grouped together for further analysis patients with overt hypogonadism (primary, secondary and normogonadotropic), as shown in Table 2.

**Table 2:** Classification of gonadal function in in a cohort of HIV-infected outpatients with hypogonadal symptoms and complete hormonal profile (Group C, n. 94)

Group C (n. 94)	Eugonadism	Compensated hypogonadism	Overt hypogonadism	P value
Anthropometric parameters				
n. (%)	62 (66.0)	13 (13.8)	19 (20.2)	**/**
Age (years)	53.5 (49.0–57.8)	52.0 (50.0–58.0)	53.0 (48.5–62.0)	0.905
BMI (kg/m^2^)	25.0 (22.0–27.4)	25.1 (22.8–26.7)	26.0 (24.1–28.9)	0.476
Hormonal parameters				
TT (ng/ml)	7.0 (5.7–9.0)	8.2 (7.0–12.0)	3.4 (2.5–4.5)	** < 0.001**
SHBG (nmol/l)	63.0 (41.8–72.8)	90.0 (61.9–98.0)	46.0 (37.0–93.0)	0.071
cFT (pg/ml)	106.5 (87.6–124.5)	89.1 (81.3–120.0)	53.7 (34.6–61.9)	** < 0.001**
LH (mUI/ml)	4.8 (3.0–7.0)	13.0 (11.0–15.9)	5.8 (3.6–10.0)	** < 0.001**
HIV parameters				
HIV infection (yr)	11.0 (9.8–20.0)	25.5 (19.0–28.5)	11.0 (6.3–16.8)	** < 0.050**
cART duration (yr)	8.0 (5.0–17.0)	17.0 (9.5–23.5)	9.5 (4–12.8)	0.084
CD4 nadir (cells/mm^3^)	148.0 (57.8–275.0)	127.0 (89.3–213.8)	63.0 (25.0–311.0)	0.798
CD4 nadir (%)	15.8 (8.1–23.8)	14.0 (12.0–15.0)	13.1 (7.5–29.3)	0.975

Categorical variables are presented as number (%). Continuous variables are expressed as median (interquartile range). Overt hypogonadism class comprises primary, secondary and normogonadotropic hypogonadism

Once again, we found, as expected, significant differences between groups for TT, cFT and gonadotropins (P < 0.001). Interestingly, SHBG values were higher in men with compensated hypogonadism with respect to the other two groups, even if this difference did not reach statistical significance. Remarkably, mean SHBG value was close to upper limit (63 nmol/l), and 34 patients (36.2%) showed values over 70 nmol/l. Among them, 18 (52.9%) were eugonadal.

No significant differences were found between groups for age, BMI, and CD4 + cell count. Conversely, we found a statistically significant difference in HIV infection duration (P < 0.05), longer in patients with compensated hypogonadism than in patients with overt hypogonadism or eugonadism. cART duration was found close to significance, again longer in patients with compensated hypogonadism than in other two classes.

**Fig. 2:**
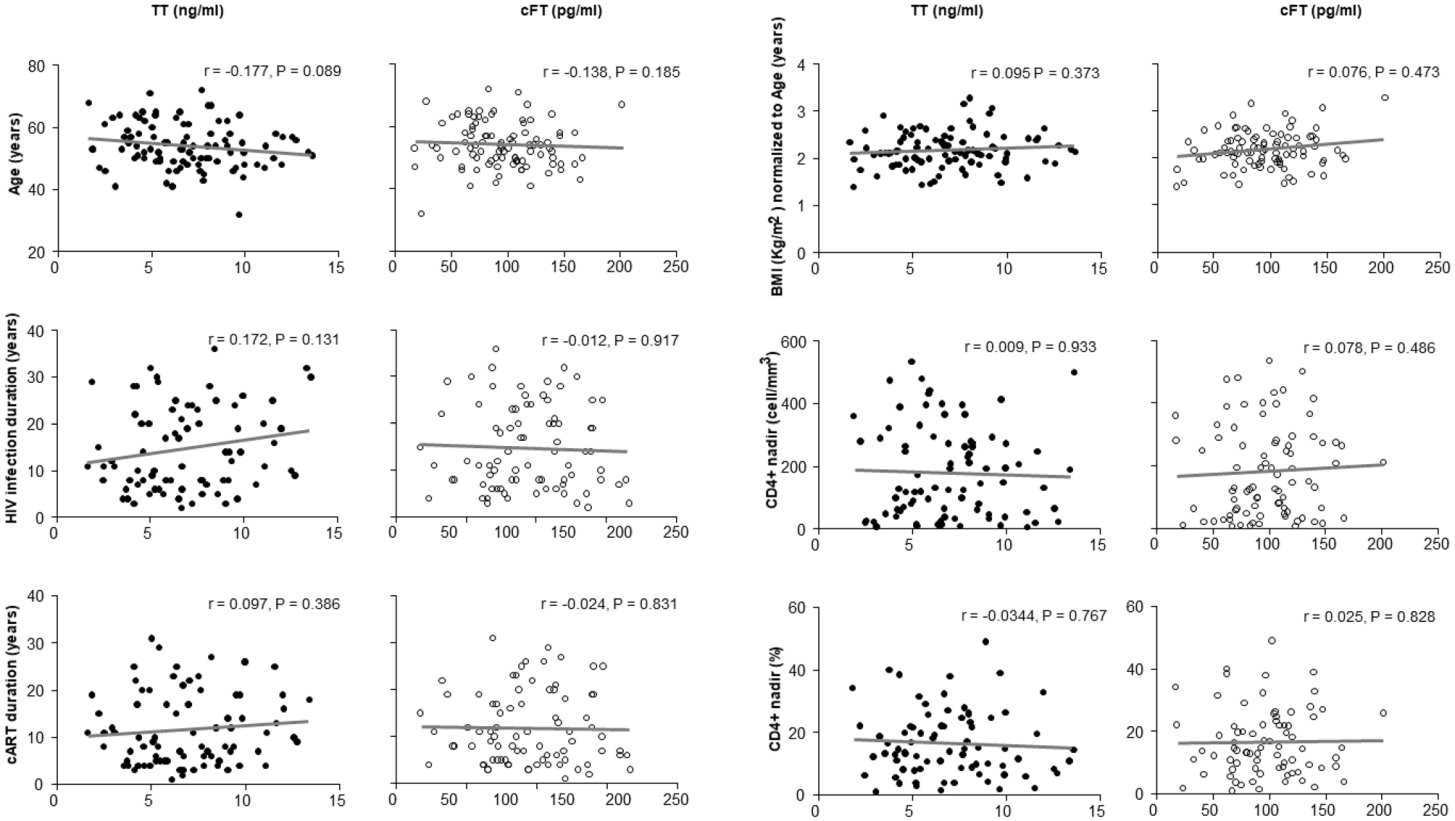
Correlations in Group C (94 patients) between clinical and virological parameters with total testosterone and calculated free testosterone

Figure 2 shows that TT and cFT did not correlate with any clinical and biochemical parameter, probably also due to the relative low number of patients. In fact, TT negatively correlated (as expected) with age and positively with duration of HIV infection when considering the whole population (n. 169, data not shown).

**Fig. 3:**
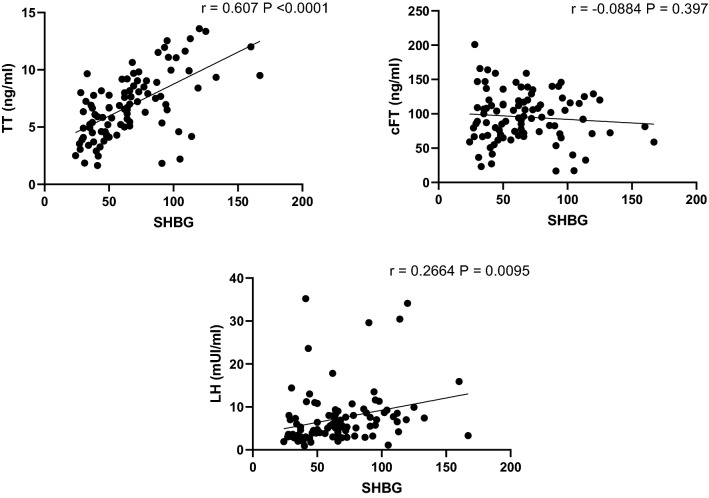
Correlation in Group C (94 patients) between total testosterone, calculated free testosterone, and LH with SHBG

Figure 3 shows the relationship between SHBG and some biochemical parameters. TT and LH showed significant positive correlation with SHBG (P < 0.0001 and P < 0.01, respectively), whereas no correlation was found between SHBG and cFT. Significant positive correlation was found between SHBG and HIV infection duration (P < 0.01), whereas none of other virological, such as cART duration and CD4 count, nor anthropometric parameters, like age and BMI, reached significance (data not shown). No significant results were found considering HBV (8/94) and HCV serostatus (33/94), smoking (39/94), alcohol (65/94) and drugs use (26/94).

## Discussion

We found overt hypogonadism, considered as the association of low TT and/or cFT levels with clinical symptoms of androgen deficit, in 20.2% of patients, in substantial agreement with previously reported data [7, 8, 13, 14, 18–21]. However, previous studies often considered only biochemical hypogonadism and the presence of symptoms was not regarded as an inclusion criterion. When considering also compensated hypogonadism, the prevalence of gonadal axis alteration in our population rose to 34.0%. Therefore, the correct diagnosis, as well as the proper classification of hypogonadism, can be made only by employing a complete hormonal profile. The use of TT alone leads to lose nearly half of diagnoses, while lack of gonadotropin measurement does not allow identifying patients with compensated hypogonadism. Such discrepancy in hypogonadism prevalence when using TT alone instead of complete hormonal profile results largely from the high SHBG values that characterize this population.

When considering biochemical definition of hypogonadism, it is essential to clarify the role of free testosterone. Basically, only 2–4% of serum testosterone circulates as free and biologically active, whereas about two thirds are tightly bound to SHBG and one third is weakly bound to albumin. This implies that in conditions with a known increase of SHBG, such as HIV infection [15], liver disease, old age, TT may be normal or even higher, but free (and, therefore, active) testosterone may be low [1, 22]. Therefore, in patients with known SHBG alteration, determination of free testosterone is mandatory. Unfortunately, direct measurement of free testosterone is unreliable [3, 9]. The gold standard is equilibrium dialysis assay, but it is quite expensive and rarely available. A commonly accepted and validate alternative is to calculate free testosterone through formulae, such as Vermeulen equation, available at http://www.issam.ch/freetesto.htm, that combine TT, SHBG and albumin [22]. Furthermore, gonadotropin levels assay allows the evaluation of the hypothalamic–pituitary–gonadal axis, to better understand etiopathogenesis of hypogonadism [1].

In our study, the use of TT alone would have resulted in a potential loss of almost 50% of diagnoses. Monroe et al*.* [8] expressed a similar concept, despite their study design was different. They found that about one-third of cases would have been misdiagnosed with reliance on TT only for hypogonadism diagnosis in spite of cFT. In our study, since Group C characteristics do not differ from Group A, it is reasonable to translate the consideration just outlined about misdiagnosis rate on the total of 169 patients. Of these 169 men, only 8.3% had low TT, but it is very likely that the addition of cFT would have doubled the cases, reaching a prevalence of about 16%, in accordance with what we found in Group C and also in literature [19].

As expected, SHBG values were generally high in our study, in accordance with literature for HIV population [13]. Higher values of SHBG were especially found in men with compensated hypogonadism, even without reaching statistical significance when compared to hypogonadal men, probably due to the relatively small number of patients included. Interestingly, we found a progressive increase in TT with HIV infection history, whereas cFT did not show significant correlation with any of the variables considered in present study. The increase in TT as the duration of infection progresses is actually misleading and depends mainly on SHBG elevation typical of these patients. In fact, there was a strong positive correlation between TT levels and SHBG, and even more importantly between SHBG and HIV infection duration, which may explain both the apparent increase of TT with infection history, and the relative stability of the cFT. Patho-physiologically, in these cases cFT is kept within normal limits by pituitary gland, which, at SHBG rising, increases secretion of LH, and, therefore, production of TT, thus establishing a new, higher equilibrium set point, to allow the maintenance of a normal free quota. Furthermore, among men with overt hypogonadism, secondary (including normogonadotropic) form was the most frequently observed, with a prevalence of 75%, in substantial agreement with literature [6], thus suggesting that these alterations may be due to a hypothalamic–pituitary–gonadal axis dysfunction, with an inadequate pituitary gland response to SHBG elevation. The finding of a relatively high prevalence of compensated hypogonadism in HIV-infected men has already been emphasized by Rochira et al*.* [14]. They suggested that this condition may precede primary gonadal failure and, therefore, should require closer monitoring due to the susceptibility to progress to overt hypogonadism. Interestingly, in our population, patients with compensated hypogonadism were also those with longer time of HIV infection and cART exposure, thus suggesting a possible slow onset gonadal compensation to infection by hypothalamic–pituitary–gonadal axis. This adaptation, however, may be very instable in time and lead to an overt hypogonadism if pituitary gland or testis stop working in synergy, thus causing secondary or primary hypogonadism, respectively. Actually, it is not well established how many patients with compensated hypogonadism may progress to overt form, even in general population [23]. However, compensated hypogonadism per se has emerged as a relevant clinical condition, associated with poor general health and cardiovascular (CV) comorbidities. A recent review [23] showed that compensated hypogonadism is associated in longitudinal studies with the incidence of major adverse cardiovascular events (MACE), the development of ED, poor health status, CV diseases and cancer. Moreover, an isolated LH elevation was found to be independently associated with overall and CV mortality and morbidity in general population [24]. Therefore, HIV- infected men with compensated hypogonadism should be carefully evaluated, and appropriately followed-up to identify the proper timing of eventual testosterone replacement therapy [5].

A strength of the present study is that all patients were symptomatic for hypogonadism (by inclusion criteria), which is hardly reported in literature to our knowledge, since only few authors considered clinical presentation of hypogonadism [13, 18, 21, 25]. This assumption is crucial, since treatment of hypogonadism is suggested only in men with both low testosterone and symptoms/signs [1, 26]. On the other side, two thirds of our patients with sexual symptoms did not have a biochemical confirmation of any form of hypogonadism, suggesting that, at least in part, these signs and symptoms can also be associated with other clinical complications and can run independently from testosterone levels. One would expect hypogonadism prevalence (low testosterone and/or high LH) to be higher among this group who was referred based on symptoms than among the total population of men in the HIV clinic. At best of our knowledge, in the only study that evaluated the prevalence of hypogonadism (based on TT levels) in HIV patients without ED *vs* those with ED [21], a prevalence of 12% vs 20% was found, respectively, similar to what we found in our paper (considering that the use of TT alone may have led to an underestimation of prevalence). Of particular note, the prevalence of symptomatic hypogonadism in our population is much higher than general population. For example, the largest study (EMAS study, 3000 subjects from Europe) performed on this topic [27] found that the association of at least three sexual symptoms with a TT level < 11 nmol/l and a FT level < 220 pmol/l was found in 2.1% of men aged 40–79 years from the general population. The prevalence increased with age from 0.1% for men 40–49 years of age, to 0.6% for those 50–59 years, to 3.2% for those 60–69 years, and to 5.1% for those 70–79 years [27].

The present study has some limitations. Firstly, it was a retrospective cross-sectional study on a relatively low number of patients, and not all patients had a complete hormonal profile. Moreover, hormonal measurements were carried out a single time and were not repeated. This is a portrait of real-life clinical practice, with a wide temporal range, and visits performed by different endocrinologists. Moreover, guidelines for hypogonadism diagnosis evolved in last years. Therefore, the lack of SHBG and/or LH measurement in some of the participants in the full sample (Group A) is due to different clinical practices and temporal changes over time, and was unlikely due to patient characteristics and does not represent a selection bias. Furthermore, due to its retrospective nature, oestradiol assay, useful to better investigate gonadal function, was not available for many patients and a control group is lacking.

In conclusion, using a complete hormonal profile, including SHBG and cFT, we found a prevalence of 20.2% of true overt hypogonadism in HIV-infected males complaining about sexual symptoms. Furthermore, gonadotropin measurement allowed us to better classify hypogonadism, and to identify compensated forms, thus increasing the prevalence of overall hypothalamic–pituitary–gonadal axis alteration to 34.0%. This finding is essential, since only a complete hormonal profile can properly recognize and classify hypogonadism in these patients. In fact, TT alone failed to diagnose half of the cases, and lack of gonadotropins prevented the identification of patients with compensated hypogonadism, which accounted for nearly 14% of the total. Although longitudinal data would better clarify some aspects on the onset and progression of hypogonadism, our data highlight some interesting pathophysiological data underlying hypogonadism in this population, especially on the role of SHBG.
